# Leptomeningeal carcinomatosis or ischemic cerebrovascular disease?

**DOI:** 10.1002/ccr3.4339

**Published:** 2021-06-04

**Authors:** Ayelet Eran, Orit Kaidar‐Person

**Affiliations:** ^1^ Neuro‐Radiology Unit Rambam Medical Center Haifa Israel; ^2^ Radiation Oncology Unit Sheba Tel Ha’shomer Tel Ha’shomer Israel

**Keywords:** brain, cerebrovascular disease, leptomeningeal, MRI

## Abstract

A lung cancer patient suspected to have leptomeningeal carcinomatosis was referred for radiation. A careful evaluation of the MRI by a neuroradiologist revealed multiple brain infarcts. Multidisciplinary meetings are important for patient's case.

## Graphical abstract

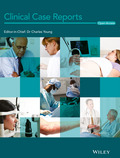

## 1 CASE

The patient was referred to the Neuro‐Oncology Unit for suspected leptomeningeal carcinomatosis. After careful evaluation of the MRI by a neuroradiology specialist at a multidisciplinary meeting, the diagnosis of multiple brain infarcts was made. Treatment with whole‐brain‐irradiation in this case would have been harmful to the patient and inappropriate.

**FIGURE 1:**
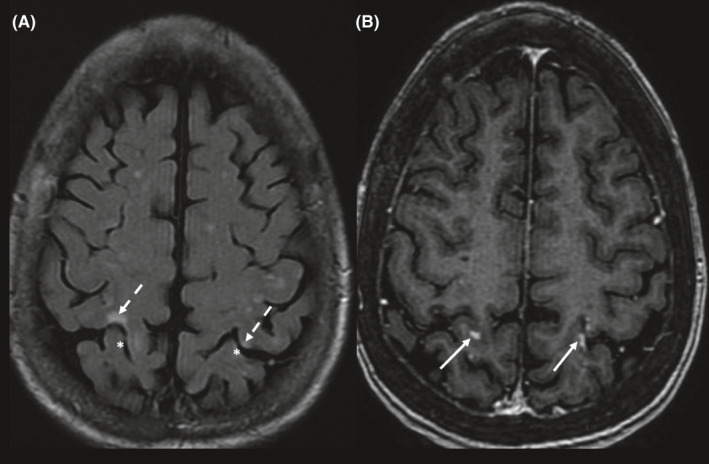
Brain MRI with gadolinium, showing cortical lesions of increased T2 signal (A) and enhancement (B, arrows). Note cortical lesion in A (dashed arrows) is none enhancing in B, while the enhancing cortical lesions in B (arrows) show almost no increased T2 signal on A (asterisks). These imaging features are inconsistent with brain metastases in which cortical T2 hyperintensity and enhancement are matching

A 82‐years‐old female, shortly after a locally advanced lung cancer diagnosis, prior to starting systemic therapy, developed right hemiparesis. Her past medical history includes a right bundle branch block and recent deep vein thrombosis. A Brain MRI with gadolinium showed scattered gyriform enhancement in supratentorial and infratentorial compartments. The lesions show increased T2 signal (Figure 1A, B) and some show diffusion restriction, no significant edema or mass effect are noted. The patient was referred to the Neuro‐Oncology Unit for suspected *leptomeningeal carcinomatosis* to consider whole‐brain irradiation. A careful evaluation of the MRI by a neuroradiology specialist at a multidisciplinary meeting revealed that not all T2 hyperintense cortical lesions were enhancing, some lesions showed diffusion restriction and lesions location matched watershed distribution. Therefore, the diagnosis of multiple brain watershed infarcts was made. The patient was referred for further evaluation for ischemic cerebrovascular disease and a follow‐up brain imaging showed improvement in the MRI abnormalities, with a resolution of enhancement, confirming the diagnosis of multiple brain infarcts. This case emphasizes the importance of multidisciplinary discussions and involving imaging experts in these meetings. Treatment with whole‐brain‐irradiation in this case would have been harmful to the patient and inappropriate.

## CONFLICT OF INTEREST

No conflict of interests or disclosures.

## AUTHOR CONTRIBUTION

Both authors contributed equally to this work and have approved the submitted version, no ethics approval was needed in this case. Their work is original and is not under consideration by any other journal.

## Data Availability

The data that support the findings of this study are available from the corresponding author upon reasonable request.

